# High genetic diversity is not essential for successful introduction

**DOI:** 10.1002/ece3.824

**Published:** 2013-10-16

**Authors:** Lee A Rollins, Angela T Moles, Serena Lam, Robert Buitenwerf, Joanna M Buswell, Claire R Brandenburger, Habacuc Flores-Moreno, Knud B Nielsen, Ellen Couchman, Gordon S Brown, Fiona J Thomson, Frank Hemmings, Richard Frankham, William B Sherwin

**Affiliations:** 1 School of Life & Environmental Sciences, Centre for Integrative Ecology, Deakin UniversityGeelong, Vic., 3216, Australia; 2 School of Biological, Earth and Environmental Sciences, Evolution & Ecology Research Centre, University of New South WalesSydney, NSW, 2052, Australia; 3 Department of Biological Sciences, Macquarie UniversitySydney, NSW, 2109, Australia

**Keywords:** Asteraceae, biological invasions, caryophyllaceae, genetic diversity, microsatellite, rapid evolution

## Abstract

Some introduced populations thrive and evolve despite the presumed loss of diversity at introduction. We aimed to quantify the amount of genetic diversity retained at introduction in species that have shown evidence of adaptation to their introduced environments. Samples were taken from native and introduced ranges of *Arctotheca populifolia* and *Petrorhagia nanteuilii*. Using microsatellite data, we identified the source for each introduction, estimated genetic diversity in native and introduced populations, and calculated the amount of diversity retained in introduced populations. These values were compared to those from a literature review of diversity in native, confamilial populations and to estimates of genetic diversity retained at introduction. Gene diversity in the native range of both species was significantly lower than for confamilials. We found that, on average, introduced populations showing evidence of adaptation to their new environments retained 81% of the genetic diversity from the native range. Introduced populations of *P. nanteuilii* had higher genetic diversity than found in the native source populations, whereas introduced populations of *A. populifolia* retained only 14% of its native diversity in one introduction and 1% in another. Our literature review has shown that most introductions demonstrating adaptive ability have lost diversity upon introduction. The two species studied here had exceptionally low native range genetic diversity. Further, the two introductions of *A. populifolia* represent the largest percentage loss of genetic diversity in a species showing evidence of substantial morphological change in the introduced range. While high genetic diversity may increase the likelihood of invasion success, the species examined here adapted to their new environments with very little neutral genetic diversity. This finding suggests that even introductions founded by small numbers of individuals have the potential to become invasive.

## Introduction

Biological invasions present one of the greatest environmental challenges of our time, yet the drivers of successful invasion remain poorly understood. The concept that genetic diversity in the founding population is positively related to the probability of invasion success is one of the oldest hypotheses in invasion biology (e.g., Mayr 1965) and continues to be supported by recent research (Crawford and Whitney 2010; Jones and Gomulkiewicz 2012). However, the debate over the importance of genetic diversity to invasion success continues (Sakai et al. 2001; Kolbe et al. 2004; Roman and Darling 2007; Hufbauer 2008). This hypothesis presents a paradox because genetic bottlenecks are expected to occur at introduction, reducing the potential for introduced populations to adapt to novel environments (Allendorf and Lundquist 2003; Allendorf and Luikart 2007), but despite this, many introduced populations thrive. In some cases, this has been explained by high levels of propagule pressure through multiple introduction events, resulting in introduced populations having greater genetic diversity than is found in the native range (Kolbe et al. 2004; Genton et al. 2005). However, successful invasions are not always accompanied by high genetic diversity and sometimes are depauperate in neutral genetic variation (Ren et al. 2005; Mergeay et al. 2006; Zimmermann et al. 2010).

Many plant and animal populations expanding into novel environments not only thrive but also exhibit rapid evolutionary changes in crucial traits such as dispersal ability, reproductive output, phenotypic plasticity, and size (Blossey and Nötzold 1995; Cody and Overton 1996; Siemann and Rogers 2001; Bossdorf et al. 2005; Phillips et al. 2006; Richards et al. 2006; Cheptou et al. 2008; Ridley and Ellstrand 2009; Buswell et al. 2011). This empirical evidence is supported by simulations, demonstrating that evolution may move at a faster rate when an organism's environment varies (Kashtan et al. 2007) and invasive populations often experience extreme environmental shifts. Further, it appears that rapid evolution in invasive species may be quite common. For example, Buswell et al. (2011) studied herbarium specimens of 23 plant species introduced to Australia and sampled repeatedly across the past ∼150 years to identify evidence of significant morphological change. They concluded that changes had occurred in 70% of these species following their introduction and that this was most likely the result of rapid evolution. Evidence of rapid evolution in novel environments supports the idea that genetic diversity is important to the success of introduced populations because adaptations following introduction are more likely to be derived from standing genetic variation rather than mutation (Barrett and Schluter 2008). Nevertheless, several studies have demonstrated rapid evolution in the presence of low genetic diversity in introduced ranges (Dlugosch and Parker 2008b; Harris et al. 2012), suggesting that the level of standing genetic diversity required for adaptation may, in fact, be quite low.

Quantitative genetic theory predicts that the extent of adaptive genetic change due to pre-existing genetic variation in the initial population in a new environment, as well as adaptation due to new mutations arising in the new environment, will be an increasing function of selection, genetic diversity, genetically effective population size, and number of generations (Robertson 1960; Weber 2004). The extent of adaptation expected for both pre-existing diversity and novel mutation can be predicted (see Appendix S1 for details), and these predictions are supported by empirical evidence (Frankham 1980b, 1983; Weber 2004; Frankham et al. 2010). As these predictions assume that genetic variation is neutral, genetic adaptation should increase with levels of neutral genetic variation, other factors being equal (Frankham et al. 1999, 2010).

In this study, we examine the relationship between neutral genetic diversity and rapid evolution in introduced species, using two species that have exhibited significant morphological change since their introduction to Australia. First, unlike many other studies of rapid evolution after introduction, we aimed to determine the exact source population(s) for the introductions for both species. This information is important for the accurate comparison of the genetic characteristics of the introduced populations from the actual source populations in their native ranges. Second, we characterized genetic diversity in the native and introduced ranges of both species. Finally, we surveyed the literature to determine (1) whether the levels of genetic diversity we found in the native range of both species were typical of other species within those families and (2) whether our species retained similar levels of genetic diversity at introduction compared with other introduced species showing evidence of rapid evolution in their introduced environments. We expected that genetic diversity in the species studied here would not be low relative to their families because they had demonstrated the ability to undergo morphological change in their introduced environments. We also expected the change in diversity between the native and introduced populations in our study species to be similar to that of other introduced species showing evidence of rapid evolution.

## Methods

### Study species

We aimed to select species that had shown potential for rapid evolution through postintroduction morphological change. We chose species with restricted native and introduced ranges so that we could comprehensively sample across their distributions. Annual or short-lived perennial species with sexual reproduction were selected, because these species have had more generations since introduction, increasing the opportunity for evolution to occur in the introduced range. We avoided selecting crop and pasture species that were likely to have been introduced many times.

**Figure 1:**
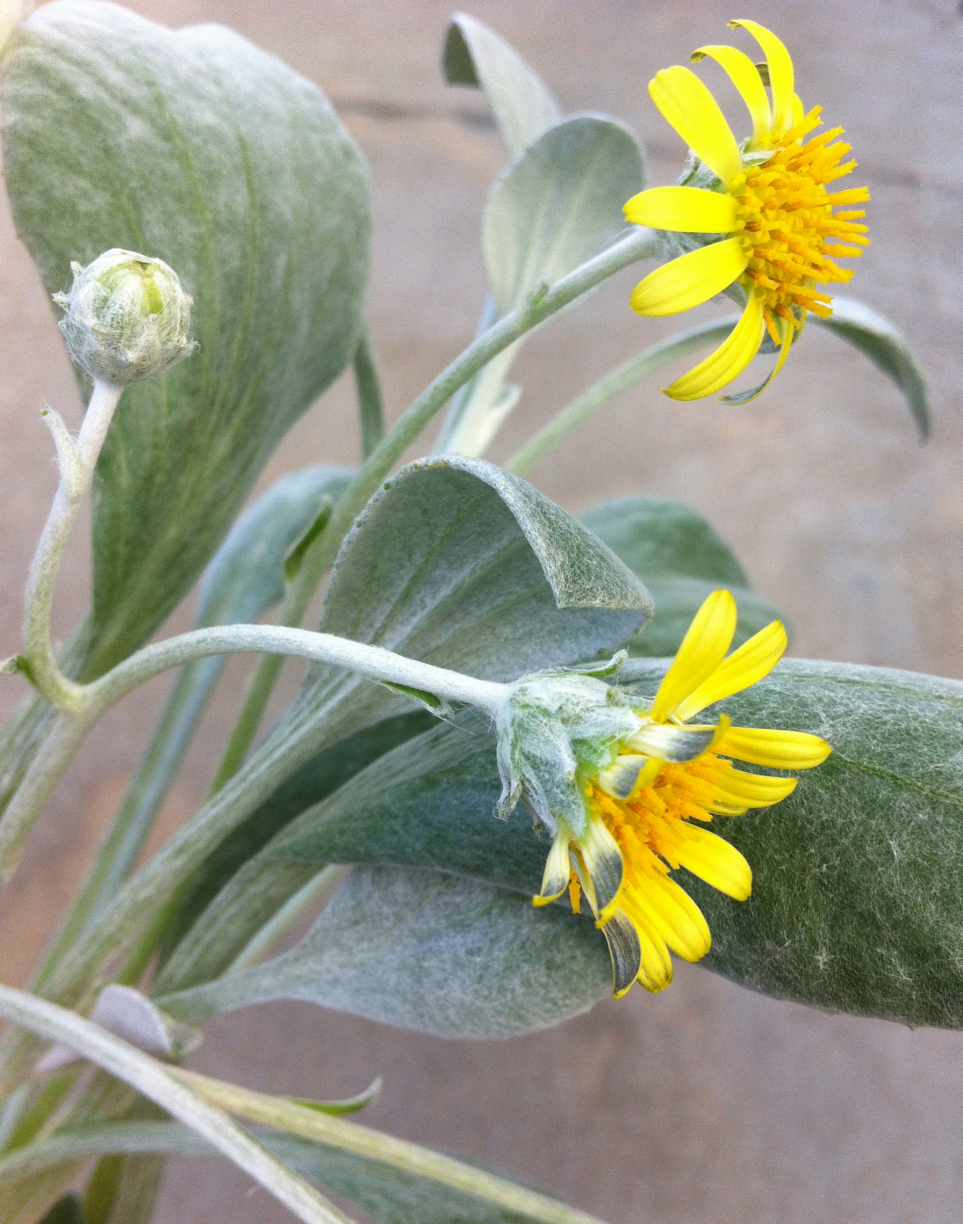
Australian sample of *Arctotheca populifolia* (photograph by C. Brandenburger).

*Arctotheca populifolia* (Fig. 1), chosen based on the findings of rapid morphological change in introduced populations (Buswell et al. 2011), is in the Asteraceae and is a perennial, herbaceous succulent native to South Africa and introduced to Australia (Harden 1992). The first records of this species in Australia occurred in the 1930s on both the east and west coasts (AVH Database, 2012). The Australian distribution encompasses coastal environments from Geraldton (Western Australia) to northern New South Wales (Heyligers 1998). The Global Compendium of Weeds lists *A. populifolia* as an agricultural and environmental weed (GCW database 2012).

The second study species was *Petrorhagia nanteuilii*, which is an annual, herbaceous plant in the Caryophyllaceae. It is native to western Europe and western North Africa (Ball and Heywood 1964) and introduced to Asia, Australia, North America, South America, and Macaronesia (GRIN database 2012). This species was first recorded in Australia in 1882, and, currently, the Australian distribution is restricted to the southeast, ranging from Brisbane to Adelaide (AVH Database, 2012). *Petrorhagia nanteuilii* is also listed in the Global Compendium of Weeds as an agricultural and environmental weed (GCW database 2012).

Similar to *A. populifolia*, *P. nanteuilii* also showed evidence of morphological change over time since introduction (see Results, below). This was determined using herbarium specimens following methods described by Buswell et al. (2011). We measured height on all available specimens at the National Herbarium of Victoria (MEL) at the Royal Botanic Gardens, Melbourne. This gave data for 184 plants from 56 herbarium sheets ranging in collection date from 1882 to 1998. No leaf traits were measured because leaves do not press well in this species. All plants had grown in the range of the introduction, in Victoria and New South Wales in Australia. We ran a general linear model including region and year as predictors and log_10_-transformed height as a dependent variable. The term for region was included to prevent the possibility that a population expansion along an environmental gradient would be mistaken for adaption to the native range across time (Buswell et al. 2011). To do this, we recorded the region of origin for each sample. Because most regions were represented by relatively few specimens, we pooled bioregions to construct four broad climate regions: (1) humid coast and hinterlands (including East Gippsland, Victoria, and the New South Wales Central Coast and South Coast), (2) humid highlands (including Eastern Highlands, the Snowfields, and the Southern Tablelands), (3) subhumid slopes (including the Victorian Midlands and Riverina, and the New South Wales South West Plains, South West Slopes, and North West Slopes), and (4) semi-Mediterranean (including the Victorian Volcanic Plain, the Grampians and Wannon). In order to acknowledge the nonindependence of plants from the same herbarium sheet, we weighted individuals according to the number measured on the herbarium sheet such that the weights for all the plants on each sheet sum to one. For example, a single plant on a sheet received a weight of one, while two individuals on the same sheet each received a weight of 0.5. Analyses were performed in JMP, version 5 (SAS Institute, Cary, NC).

For both species, we also measured plant height over time in the native range, using the methods described above. This was done in order to determine whether any changes identified in the introduced range were concurrently occurring in the native range, perhaps as a result of global climate change. For these data, region was not included as a term due to the small number of samples available for each region. In total, 52 herbarium samples from 28 sheets were measured from the native range of A. populifolia and 86 samples from 26 sheets for native range *P. nanteuilii*.

### Genetic sampling

**Figure 2:**
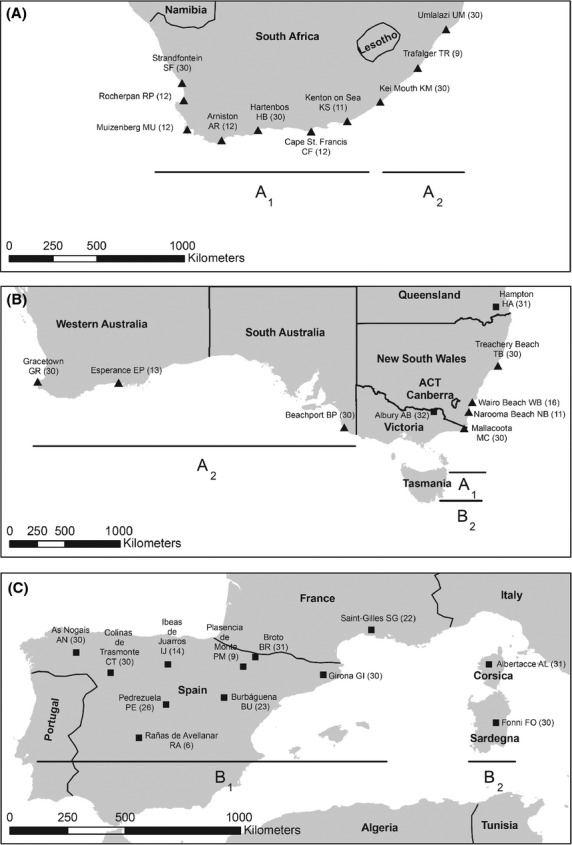
Sampled areas with place name abbreviations and number of individuals sampled in parentheses. (A) native range samples of *Arctotheca populifolia*. (B) Australian introduced range samples of *A. populifolia* (triangles) and *Petrorhagia nanteuilii* (squares). (C) native range samples of *P. nanteuilii*. Genetic groups are indicated by bars labeled with group name (i.e., A_1_; see Results). Note that group assignment of *P. nanteuilii* samples PM and GI is ambiguous (see Figs 3 and 4).

We sampled leaves from 348 *A. populifolia* plants from 10 sites covering the native range (*N* = 188; Fig. 2A) and seven sites across the introduced range in Australia (*N* = 160; Fig. 2B, triangles). For *P. nanteuilii*, we sampled a total of 345 plants, including those from 12 sites in the native range (*N* = 282; Fig. 2C) and two sites across the introduced range in Australia (Fig. 2B, squares). Two attempts were made to sample this species in the vicinity of Adelaide, South Australia, at the westernmost reported extreme of the Australian distribution, and in the vicinity of Sydney where *P. nanteuilii* has also been reported; however, on all occasions, none were present. Leaves were placed in vials containing a solution of 40% sodium chloride, 4% sodium ascorbate, 4% silica, and 3% cetyltrimethylammonium bromide (Thompson 2002) and stored at 4°C. To prepare samples for extraction, leaves were removed from the preservative, washed in Milli-Q water, patted dry, and frozen at −70°C prior to freeze drying. Freeze-dried samples were crushed and DNA was extracted using a NucleoSpin 96 Extraction II Kit (Macherey-Nagel, Düren, Germany).

Microsatellites were developed using next-generation sequencing on the GS-FLX 454 platform (Roche, Manheim, Germany) following methods described by Abdelkrim et al. (2009). QDD v 0.9.0.0 Beta (Meglécz et al. 2010) was used to identify microsatellites, and primers were designed using the program Primer 3 (Rozen and Skaletsky 2000). A panel of polymorphic markers was chosen for each species (*A. populifolia*, seven microsatellite loci; *P. nanteuilii*, 12 microsatellite loci; Table S1). Using universal primers (Neilan et al. 1997) having four differently colored fluorescent labels, we multiplexed PCRs within label color and multiloaded all loci for each species into a single reaction per individual. The step-down PCR protocol consisted of ten cycles each at the following annealing temperatures: 70^°^C, 64°C, 58°C, 54°C, 50°C. Samples were genotyped using an ABI 3730 (Applied Biosystems, Foster City, CA) using GS-500 (Liz) in each capillary as a size standard. Allele sizes were estimated on GeneMapper, version 3.7 (Applied Biosystems).

### Statistical analyses of genetic data

We tested microsatellite data for departures from Hardy–Weinberg and linkage equilibrium in Arlequin, version 3.5.1.2 (Excoffier et al. 2005), and *P*-values were Bonferroni corrected. We used Structure, version 2.2 (Pritchard et al. 2000; Falush et al. 2003), to determine whether multiple genetic groups were present across the range of each species and to determine the native source of introduced populations. For this analysis, we used the admixture model with correlated allele frequencies and tested the number of genetic groups (*K*) for each value of *K* between one and ten. We ran ten replicates for each value of *K*, each run having a burn-in period of 100,000 Markov chain Monte Carlo steps followed by 10^6^ iterations. The most likely number of genetic groups was inferred using Evanno et al.'s (2005) Δ*K* method. We determined group membership assignment of each sample using the highest proportion of membership across all ten runs of Structure. Principal coordinate analysis (PCoA) conducted in GenAlEx v. 6.3 (Peakall and Smouse 2006) was used to visualize genetic distances (Nei 1972) between populations.

Many authors have stressed that a spectrum of diversity measures gives the best summary of diversity (Pielou 1966; Hill 1973). Therefore, we used measures closely related to each of Hill's first three diversity orders: zero (number of alleles, *NA*; allelic richness, *R*), unity (Shannon's Index, ^*S*^*H*), and two (Hardy–Weinberg expected heterozygosity, *H*_E_). To calculate *NA*, *R,* and *H*_E_ for each sample, we used FSTAT, version 2.9.3.2 (Goudet 1995, 2002), and ^*S*^*H* was calculated using GenAlEx. For greatest utility in future comparisons, we also convert diversity orders 1 and 2 into their effective number equivalents, which avoid many well-known problems of diversity measures (Jost et al. 2010; Leinster and Cobbold 2011). The respective effective numbers equivalents are ^1^D_within_ = 2∧^*S*^*H*, and ^2^D = 1/(1−*H*_E_).

We used nonparametric Mann–Whitney *U-*tests to compare within-population diversity levels between the samples identified as sources for Australian introductions of both species because these data could not be made normal by transformation. Three approaches were used to assess diversity between populations. Pairwise *F*_ST_ values were calculated in Arlequin for comparison with other studies that quote this measure. Pairwise values for Shannon's mutual information index (^*S*^*H*_UA_) were calculated in GenAlEx. Compared with *F*_ST_, mutual information is known to be more robust to a wide range of population sizes and dispersal rates (Sherwin et al. 2006; Dewar et al. 2011); additionally, the mutual information index can be converted to a numbers equivalent (^1^D_between_), which avoids some serious problems that occur with other between-population measures (Jost et al. 2010).

We surveyed the literature regarding *H*_E_ measured from polymorphic microsatellite data in species from both families containing our study taxa, Asteraceae and Caryophyllaceae, to determine whether *H*_E_ estimates generated from native populations in this study were congruent with those from other members of the same family. This search was conducted in Google Scholar using the family name as a search term in conjunction with the terms “microsatellite” and “heterozygosity” in August 2012. Where data were given for multiple populations within a study, a mean value of *H*_E_ was used. We avoided including estimates generated from introduced ranges, those of populations suspected of hybridization, and those of cultivated populations. Then, we surveyed the literature for examples of species showing evolutionary change in their introduced range, where genetic diversity had been estimated in both the native and introduced ranges. This search was conducted in Google Scholar in October 2012 using the terms “introduced” and “heterozygosity” in conjunction with either “rapid evolution” or “contemporary evolution.” Additionally, we included studies referenced in a review of genetic variation across native and introduced ranges (Dlugosch and Parker 2008a) showing evidence of morphological change in the introduced environment. We calculated the ratio of diversity found in the introduced range to that found in the native range (^*R*^*H*_E_), which gives an estimate of diversity retained after introduction, assuming no changes in diversity have occurred in the introduced range. For this calculation, we only used estimates of *H*_E_ generated from microsatellite data because the absolute values of diversity estimates differ according to the marker used, and we wanted to directly compare these results to those generated in the current study.

## Results

### Evidence of morphological change

**Figure 3:**
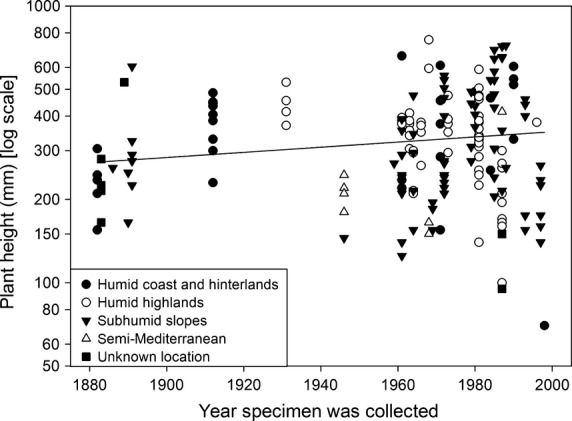
Log_10_ plant height of *Petrorhagia nanteuilii* introduced to Australia measured from herbarium specimens sampled from 1880 to 2000, classed by climatic region. Values increased significantly across time (weighted general linear model including a term for region; *R*^2^ = 0.06; *F*_year1,176_ = 5.06; *P*_region_ = 0.13; *P*_year_ = 0.026).

*Petrorhagia nanteuilii* in the introduced range showed a significant increase in log_10_ height through time that was not driven by region sampled (weighted general linear model including a term for region; *R*^2^ = 0.06; *F*_year1,176_ = 5.06; *P*_region_ = 0.13; *P*_year_ = 0.026; Fig. 3). Although the predictive power of this relationship is low, the magnitude of change is high, with average plant height increasing by almost 30% between 1882 and 1998.

In the native range of *A.populifolia*, log_10_ plant height was unchanged across the period of this study (weighted general linear model; *R*^2^ = 0.003; *F*_year1,52_ = 0.15; *P*_year_ = 0.70; Fig. S1a). In the native range of *P. nanteuilii*, log_10_ plant height decreased through time (weighted general linear model; *R*^2^ = 0.05; *F*_year1,85_ = 3.98; *P*_year_ = 0.049; Fig. S1b).

### Microsatellite markers

We found no evidence for departures from Hardy–Weinberg and linkage equilibrium in the microsatellite data for *A. populifolia*. Two of the twelve loci developed for *P. nanteuilii* (*Pna06* and *Pna16*) significantly deviated from Hardy–Weinberg equilibrium and were excluded from downstream analyses. The remaining ten loci showed no significant departures from equilibrium. *Petrorhagia nanteuilii* has previously been reported to be tetraploid (Thomas and Murray 1981). Although we found no evidence of tetraploidy in the microsatellite data presented here, it is possible that in allotetraploid species, only a single parental genome may be amplified from any pair of primers.

### Population structure

**Figure 4:**
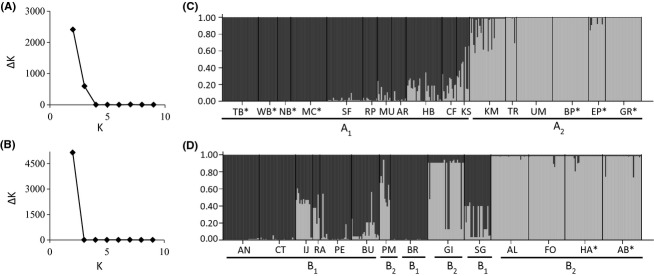
Structure analyses. Evanno et al.'s (2005) Δ*K* values for each putative number of populations (*K*) for (A) *Arctotheca populifolia* and (B) *Petrorhagia nanteuilii*. Structure Q plots generated using the maximum value of Δ*K* indicate *A. populifolia* (C) and *P. nanteuilii* (D) samples represent two genetic groups each, demarcated by labeled bars (i.e., A_1_). Samples from the introduced range are denoted by asterisks. Each individual is represented by a vertical line showing degree of admixture. Sample name abbreviations are defined in Figure 1.

The Structure analysis of *A. populifolia* suggested the presence of two genetic groups (Fig. 4). One group included native samples extending from the western edge of the range in South Africa to Kenton on Sea as well as samples from south-eastern Australia (Fig. 4, group A_1_). The second group contained native samples from the eastern edge of the range to Kei Mouth and also included samples from Western Australia (Fig. 4, group A_2_). Two genetic groups were identified in *P. nanteuilii*: One group consisted of most sampling localities in Spain and France (Fig. 4, group B_1_) and a second group (Fig. 4, group B_2_) contained samples from two localities in Spain (Plasencia del Monte, PM; and Girona, GI), Corsica, Sardinia, and Australia. The samples from Corsica, Sardinia, and Australia had membership proportions for group B_2_ in excess of 0.97, whereas those from Spain were lower (PM, B_2_ membership proportion = 0.61; GI, B_2_ membership proportion = 0.80).

**Figure 5:**
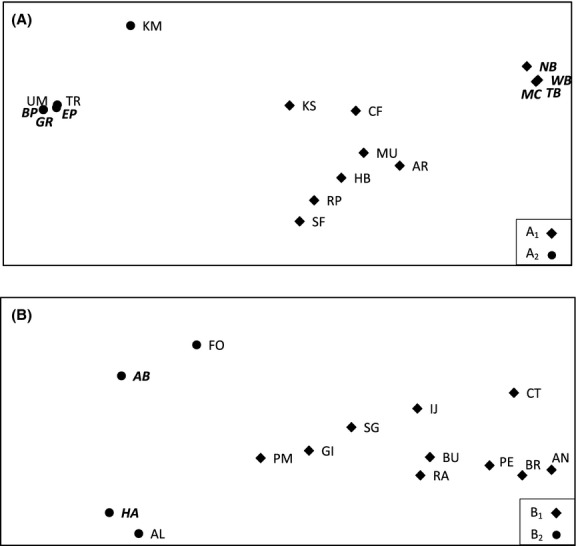
Principal coordinates analysis of genetic distance between *Arctotheca populifolia* samples (A) and *Petrorhagia nanteuilii* samples (B). Genetic groups identified in Structure analyses denoted by diamonds (A_1_ and B_1_) and circles (A_2_ and B_2_). Sample name abbreviations are defined in Figure 1 and those in bold represent introduced samples.

Principal coordinate analysis plots were generally concordant with Structure results. *Arctotheca populifolia* samples from group A_1_ (Fig. 5A, diamonds) formed two distinct clusters representing native and introduced populations, respectively. A_1_-introduced samples were more closely related to A_1_ native samples than any A_2_ samples (Fig. 5A, circles). Within the A_2_ group, the Kei Mouth sample was separated from all other samples. The remaining native A_2_ samples were clustered with introduced A_2_ samples. *Petrorhagia nanteuilii* samples from group B_1_ (Fig. 5B, diamonds) were clustered together. The two Spanish samples that had lower membership proportions for group B_2_ in the Structure analysis were clustered with samples in group B_1_. Group B_2_ included the introduced sample from Hampton, which was clustered with the native sample from Corsica, and the introduced sample from Albury, which clustered with the native sample from Sardinia (Fig. 5B, circles).

### Genetic diversity

In the introduction to eastern Australia, native samples of *A. populifolia* had significantly higher *H*_E_ (Table 1) than did introduced samples (A_1_ introduction: Mann–Whitney *U*, *P <* 0.01), while in the introduction to Western Australia, *H*_E_ was not different between native and introduced samples (A_2_ introduction: Mann–Whitney *U*, *P* = 0.70). Diversity was low within the second introduction: We found a single genotype across all seven loci in one *A. populifolia* sample from the northeastern extreme of the native range (Umlalazi) and three samples from Australia (Beachport, Narooma and Wairo Beach). Assuming a single introduction of *P. nanteuilii* to Australia, the estimated *H*_E_ in the native samples was not different to that of introduced samples (B_2_ introduction: Mann–Whitney *U*, *P* = 0.33). Values of *R* were similarly low in both species (Table 1). Despite the low values of genetic diversity, we detected that within populations, the total number of alleles detected for each species was not particularly low; 36 alleles were detected across the seven loci used for *A. populifolia* and 50 alleles across the ten loci used for downstream analysis in *P. nanteuilii*. This highlights the strong genetic diversity found between samples across the native ranges of both species (Table 1; *F*_ST_, 0.33–0.56; ^*S*^*H*_UA_, 0.09–0.33).

**Table 1:** Estimation of diversity within native and introduced populations of *Arctotheca populifolia* and *Petrorhagia nanteuilii* including measures across three diversity orders: zero (allelic richness, *R*), unity (Shannon index, ^*S*^*H*; and the effective numbers equivalent, ^1^D_within_), and two (Hardy–Weinberg expected heterozygosity, *H*_E_; and the effective numbers equivalent, ^2^D). For both species, genetic differentiation within the native and introduced ranges was calculated using *F*_ST_ and Shannon's mutual information index (^*S*^*H*_UA_), and the numerical equivalent of ^*S*^*H*_UA_ (^1^D_between_)

	*Arctotheca populifolia* (A_1_)		*Arctotheca populifolia* (A_2_)		*Petrorhagia nanteuilii* (B_2_)	
			
Statistic	Native	Introduced	Native	Introduced	Native	Introduced
Mean *R* (range)	2.2 (2.0–2.6)	1.0 (1.0–1.1)	1.3 (1.0–1.8)	1.1 (1.0–1.2)	1.1^1^	1.4 (1.3–1.4)
Mean ^*S*^*H* (range)	0.79 (0.57–0.96)	0.08 (0.0–0.30)	0.18 (0.0–0.46)	0.04 (0.0–0.09)	0.09 (0.08–0.11)	0.33 (0.29–0.38)
Mean ^1^*D*_within_ (range)	1.73 (1.48–1.94)	1.06 (1.0–1.23)	1.14 (1.0–1.38)	1.03 (1.0–1.06)	1.07 (1.05–1.08)	1.26 (1.22–1.30)
Mean *H*_E_ (range)	0.34 (0.23–0.43)	<0.01 (0.0–0.01)	0.07 (0.0–0.18)	0.01 (0.0–0.03)	0.04 (0.02–0.05)	0.13 (0.11–0.15)
Mean ^2^*D* (range)	1.53 (1.31–1.74)	1.01 (1.0–1.01)	1.09 (1.0–1.22)	1.01 (1.0–1.03)	1.04 (1.02–1.06)	1.15 (1.12–1.18)
Mean Pairwise *F*_ST_ (range)	0.33 (0.09–0.56)	<0.01 (0.0–0.01)	0.40 (0.19–0.59)	0.05 (0.02–0.08)	0.56 (0.18–0.77)	0.52^2^
Mean Pairwise ^S^H_UA_ (range)	0.23 (0.08–0.41)	<0.01 (0.0–0.01)	0.09 (0.01–0.16)	0.01 (0.01–0.002)	0.33 (0.27–0.41)	0.26^2^
Mean ^1^*D*_between_ (range)	1.18 (1.06–1.33)	1.00^1^	1.06 (1.01–1.12)	1.01 (1.00–1.01)	1.25 (1.21–1.33)	1.20^2^

1 All measures equal mean.

2 Denotes a single pairwise comparison.

**Figure 6:**
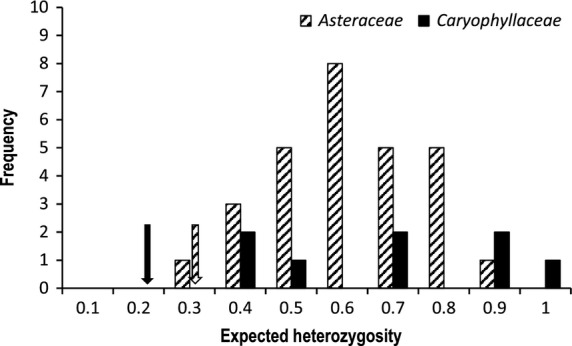
Native range estimates of expected heterozygosity from microsatellite data in Asteraceae (striped) and Caryophyllaceae (solid) families (see Table S1, for details). Arrows indicate the level of heterozygosity found in the native ranges of the species used in the present study (calculated from all sites sampled in the native range of each species).

We found microsatellite estimates of *H*_E_ in 28 Asteraceae species from 28 different genera ranging from 0.22 to 0.88 (mean *H*_E_ = 0.58, Fig. 6 and, Table S2). Of the approximately 2200 species in Caryophyllaceae (Schweingruber et al. 2011), microsatellite data exist for only eight species from five genera. We used expected heterozygosity measures from all eight of these species ranging from 0.35 to 0.93 (mean *H*_E_ = 0.64, Fig. 6 and Table S2). In this study, values of *H*_E_ across the native ranges of *A. populifolia* and *P. nanteuilii* were lower than almost all values we found for confamilials (Fig. 6; *A. populifolia H*_E_ = 0.26; *P. nanteuilii H*_E_ = 0.17) and both were significantly lower than the mean of these values for each family (one-sample *t*-tests: *A. populifolia*, *P* < 0.001; *P. nanteuilii*, *P* = 0.001).

We found 19 examples in the literature of species that had demonstrated evolutionary change in their introduced environment and where genetic diversity had been estimated in both the native and introduced range. Eight of these studies used allozymes, half of which found higher genetic diversity in the native range (Table 2). Of the eleven studies that used microsatellites, only one estimated a higher genetic diversity in the introduced range as compared to the native range. Ten of the microsatellite studies could be directly compared to our results (i.e., they provided estimates of *H*_E_). In these studies, the ratio of diversity in the introduced range to the diversity in the native range was an average of 0.81 (^*R*^*H*_E_ range: 0.30–1.22, Table 2). In the present study, more diversity was found in the introduced range than the native source population of *P. nanteuilii* (^*R*^*H*_E_ = 3.25). However, ^*R*^*H*_E_ for *A. populifolia* was much lower than estimates found in the literature search (A_1_ introduction: ^*R*^*H*_E_ = 0.01, A_2_ introduction: ^*R*^*H*_E_ = 0.14). In fact, both introductions of *A. populifolia* had the lowest ratios of any example we found.

**Table 2:** Species showing evidence of evolutionary change in introduced environments and for which genetic diversity was measured in native and introduced populations. The ratio of genetic diversity in the introduced range to the native range is given, and the direction of change is given (Trend). The statistics used to calculate diversity included allelic richness (*R*), expected heterozygosity (*H*_E_) and genet richness (*GR*). Effective number equivalents (E) have been calculated for native/introduced diversity

	Genetic Diversity						
						
Species	Native	Introduced	Ratio	Trend	Statistic	E	Reference
Allozymes							
*Acridotheres tristis*	0.06	0.03	0.50	−	*H*_E_	1.06/1.03	Baker and Moeed (1987), Berthouly-Salazar et al. (2012)
*Bufo marinus*	0.39^1^	0.36	0.91	−	*H*_E_	1.64/1.56	Estoup et al. (2001), Phillips et al. (2006)
*Cedrus atlantica*	0.19	0.16	0.88	−	*H*_E_	1.23/1.20	Bariteau and Ferrandes (1992), Lefevre et al. (2004)
*Clidemia hirta*	0.04	0.06	1.40	+	*H*_E_	1.04/1.06	DeWalt et al. (2004), DeWalt and Hamrick (2004)
*Fringilla coelebs*	0.05	0.07	1.40	+	*H*_E_	1.05/1.07	Baker (1992)
*Gambusia affinis*	0.14	0.15	1.07	+	*H*_E_	1.16/1.17	Stearns (1983), Scribner et al. (1992)
*Passer montanus*	0.10	0.08	0.77	−	*H*_E_	1.11/1.08	Barlow (1980), St. Louis and Barlow (1988)
*Phalaris arundinacea*	1.89	2.27	1.20	+	*R*	1.89/2.27	Lavergne and Molofsky (2007)
		Mean^2^	0.99				
Microsatellites							
*Alliaria petiolata*	0.22	0.12	0.55	−	*H*_E_	1.28/1.14	Durka et al. (2005), Bossdorf et al. (2004a,b)
*Ambrosia artemisifolia*	0.76	0.75	0.99	−	*H*_E_	4.10/3.94	Genton et al. (2005), Hodgins and Rieseberg (2011)
*Carpodacus mexicanus*	0.81	0.77	0.95	−	*H*_E_	5.24/4.37	Able and Belthoff (1998), Egbert and Belthoff (2003), Hawley et al. (2006)
*Coregonus albula*	0.60	0.73	1.22	+	*H*_E_	2.47/3.65	Amundsen et al. (2012)
*Drososphila suboscura*	0.87	0.70	0.80	−	*H*_E_	7.94/3.33	Huey et al. (2000), Pascual et al. (2001)
*Linepithema humile*	0.64	0.20	0.31	−	*H*_E_	2.78/1.25	Tsutsui et al.(2000)
*Microstegium vimineum*	0.24	0.16	0.67	−	*H*_E_	1.32/1.19	Novy et al. (2012a,b)
*Oryctolagus cuniculus*	0.69	0.67	0.97	−	*H*_E_	3.23/3.03	Williams and Moore (1989), Zenger et al.(2003)
*Phragmites australis*	0.74	0.22	0.30	−	*GR*	0.74/.022	Saltonstall and Stevenson (2007), Kettenring and Mock (2012)
*Rhagoletis completa*	0.52	0.50	0.96	−	*H*_E_	2.08/2.00	Chen et al. (2006)
*Thymallus thymallus*	0.19^1^	0.13	0.68	−	*H*_E_	1.23/1.15	Koskinen et al. (2002)
		Mean^2^	0.81				
*Arctotheca populifolia*							
A_1_ introduction	0.34	<0.01	0.01	−	*H*_E_	1.53/1.01	This study
A_2_ introduction	0.07	0.01	0.14	−	*H*_E_	1.09/1.01	This study
*Petrorhagia nanteuilii*							
B_2_ introduction	0.04	0.13	3.25	+	*H*_E_	1.04/1.15	This study

1 These estimates are from primary introductions, which were the sources of secondary introductions (“introduced” values for these species).

2 Mean includes all species having *H*_E_ estimates.

## Discussion

Genetic diversity has been demonstrated to be positively correlated with invasion success (Crawford and Whitney 2010), and standing genetic variation is believed to be important to invasive species' ability to adapt to novel environments (Barrett and Schluter 2008). However, it is becoming clear that introduced populations with very low neutral genetic diversity are sometimes successful invaders (Ren et al. 2005; Mergeay et al. 2006; Zimmermann et al. 2010) and have the ability to adapt to their new environments (Dlugosch and Parker 2008b; Harris et al. 2012). Here, we provide two examples of species that have established, spread, and adapted to the environment in their introduced range in Australia (Buswell et al. 2011 and Fig. 3), yet have significantly lower genetic diversity in their native ranges than do confamilials. Interestingly, for both of these species, the changes we identified in the introduced ranges were not found in the native ranges, ruling out the possibility that global processes are driving these changes.

Despite the fact that the two separate introductions of *A. populifolia* described here had comparatively low levels of genetic diversity in the native source populations, these two introductions represent a larger percentage loss of genetic diversity than found in any introduction identified in our review of species, showing substantial morphological change in the introduced range. Similarly, introduced populations of invasive Japanese knotweed (*Fallopia* species complex) harbored very low genetic diversity at Amplified Fragment Length Polymorphism markers despite displaying significantly different phenotypes in a common garden setting (Richards et al. 2008). While greater levels of diversity may increase the likelihood of invasion success (Crawford and Whitney 2010; Jones and Gomulkiewicz 2012), it is clear that some introduced species, such as those discussed here, are able to become invasive and adapt to their new environments with very little neutral genetic diversity. This has important management implications because it demonstrates that even introductions from very small numbers of individuals have the potential to become invasive.

The review we have conducted specifically examines loss of genetic diversity at introduction in species where some evidence of adaptive change has been documented in the introduced environment. It would be useful to compare the associated change in genetic diversity in this group with that of a group of species which has been introduced but has shown no evidence of adaptation to novel environments; we might anticipate that the latter would show more loss if diversity is important to adaptive potential. Unfortunately, there is a bias in reporting which makes this difficult. However, we can compare the results of our review (19% loss of GD in the introduced range) with that of Dlugosch and Parker (2008a), who found 22.6% loss of diversity at introduction irrespective of evidence of adaptive change (Dlugosch and Parker 2008a) (two-sample *t*-test, *P* = 0.72). This suggests that neutral genetic diversity is not important to adaptive potential in introduced species.

The ability of populations with low current “neutral” diversity to evolve could be due to (1) retention of greater adaptive than neutral genetic variation due to either chance or balancing selection on adaptive variation (Reed and Frankham 2001), (2) contributions of mutations to selection response, especially when selection lasts for more than twenty generations (Frankham 1980b, 1983; Hill 1982a,b), (3) loss of neutral genetic diversity after much of the adaptation has occurred, or (4) some combination of these. Substantial adaptive genetic changes can still occur in populations subject to bottlenecks, and there is often large variation among replicates (Frankham 1980a). We are unable to distinguish between these hypotheses, but the likelihood of contributions from mutations that arose after introduction increases as the level of neutral genetic diversity in the introduced population decreases.

Recent research suggests that epigenetic modifications (DNA methylation) may also play an important role in invasion success. Using the invasive Japanese knotwood populations discussed above, Richards et al. (2012) demonstrated that although genetic diversity was extremely low, significant epigenetic differentiation occurred between sites, suggesting a possible nongenetic mechanism for adaptation. Theoretical work on epigenetic selection models indicates that increased phenotypic change can occur in populations with no genetic variation as a result of epigenetic changes (Geoghegan and Spencer 2012). In fact, Liebl et al. (2013) found a negative relationship between genetic and epigenetic diversity in introduced populations of sparrows (*Passer domesticus*) and speculated that epigenetic variation may provide a mechanism for adaptation over the short time scales relevant to invasions.

### Source population identification

Our results highlight the importance of determining source populations prior to assessing changes in genetic diversity between native and introduced ranges. Few studies investigating this topic have done this, but levels of genetic diversity can be very different across a species' native range, as we found with *A. populifolia*. Identifying the source of an invasion assures that observed differences between introduced and native populations are not the result of diversity within the native range and prevents actual differences from becoming obscured (Dlugosch and Parker 2008a). Similarly, for studies attempting to identify contemporary evolution in introduced species, it is vital that the correct source population is identified in the native range.

Our analyses identified two genetic groups of *A. populifolia*. One group consisted of western South African native samples and eastern Australian introduced samples. PCoA indicated that the latter were most similar to native samples from the south coast of South Africa ranging from Muizenberg to Kenton on Sea. The second group contained samples from the east coast of South Africa and introduced samples ranging from Margaret River in Western Australia to Beachport in South Australia. The two genetic groups identified in South Africa correspond perfectly to the Cape Seashore Vegetation (Group A1) and the Subtropical Seashore Vegetation (Group A2) described by Mucina and Rutherford (2006). A single genotype across seven loci was found in all 30 individuals sampled at Umlalazi (eastern South Africa), and this genotype was found in every individual sampled in Beachport and 39 of the 43 individuals sampled in Western Australia. These results indicate two separate introductions to Australia. This is supported by morphological data indicating that two forms of *A. populifolia* exist in Australia, both of which are found in South Africa (Heyligers 2007). The Victorian coastline separates the two morphological groups (Heyligers 2007) as well as the genetic groups found in this study.

Samples within native populations of both species were highly differentiated. This is possibly due to our intentional selection of species with restricted ranges so that we could sample comprehensively across the native ranges. Although restricted ranges and high levels of population differentiation can be caused by limited dispersal, there is independent evidence that there may be a small amount of long-distance dispersal in *A. populifolia*. Heyligers (2007) argued that the distribution of *A. populifolia* morphs in Australia could be explained by dispersal of achenes via coastal currents and that historical records of first appearance showed an eastward progression of this introduction from Western Australia to South Australia. Given this evidence, one might expect to find a more cosmopolitan distribution of this species, but to our knowledge, *A. populifolia* is only found in southern Africa and Australia.

The Structure analysis of *P. nanteuilii* indicated that two genetic groups exist in the native range, but only one of these was represented within Australia. Mediterranean island samples from Corsica and Sardinia were most similar to samples from Australia, supporting a single source for this introduction. However, while the PCoA supported the membership of group B_1_ determined in Structure (Fig. 5B), the samples contained within group B_2_ were not well clustered. In fact, the PCoA indicated that the introduced sample from Hampton was closely related to the native sample from Corsica, whereas the introduced sample from Albury was closely related to the native sample from Sardinia. This raises the possibility that two introductions of this species into Australia may have occurred.

### Comparisons of genetic diversity between introduced and native ranges

Low genetic diversity in introduced populations can reflect a genetically impoverished source (Voss et al. 2012). Our identification of the source populations for the introductions discussed here allows us to confirm that the sources were genetically impoverished. Within native and introduced populations of *A. populifolia* and *P. nanteuilii,* values of *H*_E_ were considerably lower than found in other species within these families (Table 1; Fig. 6; Table S2). One native sample of *A. populifolia* displayed no gene diversity (i.e., had a single genotype across seven loci). Variation was even lower in the introduced range of *A. populifolia* than the already low variation in the native range, suggestive of a small number of founders.

Despite low levels of within-population neutral genetic diversity, both of these species are widespread in their introduced range in Australia. Similarly, Hardesty et al. (2012) found extremely low levels of neutral genetic variation in highly successful introductions of *Miconia calvescens* (mean *H*_E_ = 0.07). However, because the ability to evolve in response to a novel environment may depend on the level of adaptive variation present, the relevance of neutral markers variation has been questioned (Reed and Frankham 2001). Although a positive correlation has been reported between quantitative trait (*Q*_ST_) and microsatellite variation (*F*_ST_), quantitative variation is usually higher (Merilä and Crnokrak 2001; Leinonen et al. 2008) and the ability to predict *Q*_ST_ increases with increasing values of *F*_ST_ (Leinonen et al. 2008). Population bottlenecks are predicted to reduce additive genetic variance (Wright 1951; Chakraborty and Nei 1982), but in some circumstances, such populations may experience an increase in additive genetic variance for traits with at least some nonadditive genetic variation (Willis and Orr 1993; Wang et al. 1998; Willi et al. 2006). While this shift in additive genetic variance may not always result in an increased ability to adapt to novel selection pressures (van Heerwaarden et al. 2008), the combination of adaptation with low diversity at neutral markers indicating the presence of a bottleneck has been identified here and in other studies (Koskinen et al. 2002; Yonekura et al. 2007; Dlugosch and Parker 2008b). Frankham et al. (1999) showed that the effects of population bottlenecks on ability to evolve in response to environmental change closely followed neutral expectations. Further, a number of data sets indicate that genetic variation involved in adaptation to new environments is approximately additive (de Oliveira and Cordeiro 1980; Frankham et al. 1999), in contrast to the fitness variation in the environment to which populations have been adapted long-term, where there is usually a predominance of nonadditive variation (i.e., the occurrence of increased additive genetic variation in bottlenecked populations probably does not apply to populations adapting to new environments).

Finally, we speculate on whether intrinsic characteristics of the species might be affecting the diversity. Finding a genetically monomorphic sample in the native range may suggest alternate forms of reproduction across the species' distribution. When Roman and Darling (2007) examined successful introductions having decreased genetic diversity in the introduced range, 63% had reproductive abilities other than those involving sexual recombination. It is possible that *A. populifolia* may have the ability to reproduce via apomixis, spread vegetatively, or self-fertilize, although none of these reproductive mechanisms have been reported in this species. *Arctotheca populifolia* has previously been reported as diploid (Norlindh 1967), and our investigations of ploidy in both native and introduced samples support this (see Appendix S2). This suggests that apomixis is an unlikely explanation for the observed genetic pattern because, among Asteraceae, this reproductive mechanism is normally only found in polyploids (Noyes 2007). Vegetative reproduction has been reported in the congener *A. calendula* (Bossard 2000), but does not explain the biogeographic patterns of *A. populifolia* described here. Baker's Law (Stebbins 1957) states that self-fertilization should provide an advantage to colonizing populations (Baker 1955) and could explain the genetic patterns we have identified in eastern South Africa and Australian introduced populations. Baker's Law is supported by three findings: i) increased frequency of self-compatible species on islands (Barrett et al. 1996), ii) species capable of autonomous seed production had larger invasive ranges (van Kleunen and Johnson 2007), and iii) species naturalized outside of their native range are more likely to self-fertilize than congeners only found in their native range (van Kleunen et al. 2008).

## Conclusion

Considerable effort has been invested in identifying drivers of invasion success, including the importance of genetic diversity to invasiveness. While genetic diversity may be related to invasion success in some species (Crawford and Whitney 2010), increasingly, evidence suggests that genetic diversity is not essential to a species' ability to invade novel environments. Here, we have identified two species with low levels of neutral genetic variation in both their native and introduced ranges, which appear to have adapted and spread in their introduced range. Recent empirical evidence and simulations suggest that a number of factors influence the relationship between genetic diversity and invasion success and that complexities such as competitive interactions and diversity of the native community are likely to be important (Chang and Smith 2012; Hovick et al. 2012; Jones and Gomulkiewicz 2012). Further, it appears that epigenetic modifications may play a role in facilitating invasion success immediately following invasion, although this idea has not yet been rigorously tested. In combination, these results suggest that genetic diversity measures alone are inadequate predictors of invasion success.

## Supporting Information

**Appendix S1.** Theory of adaptive genetic change in novel environments.

**Appendix S2.** Chromosome investigations of *Arctotheca populifolia*.

**Table S1.** Characterization of microsatellite loci in *Arctotheca populifolia* (*N* = 348) and *Petrorhagia nanteuilii* (*N* = 377) including locus name, GenBank accession number, primer sequences, repeat motif, number of alleles and allele size range.

**Table S2.** Expected heterozygosity (*H*_E_) estimates from microsatellite data for species within the Asteraceae and Caryophyllaceae families. Effective number equivalents (^2^*D*) have been calculated. For Asteraceae, estimates were included for one species per genus (*N* = 28). For Caryophyllaceae, all estimates identified in the literature were included (*N* = 8). The number of loci (L), samples (S) and total number of individuals (I) are given for each study.

**Figure S1.** (A,B) Plant height (log_10_ transformed) of *Arctotheca populifolia* (A) and *Petrorhagia nanteuilii* (B) measured from herbarium specimens sampled in the native range between 1891–2003 and 1848–1985, respectively. For *A. populifolia*, values do not change significantly over this time period (weighted general linear model; *R*^2^ = 0.003; *F*_year1,52_ = 0.15; *P* = 0.70). For *P. nanteuilii*, plant height decreased through time (weighted general linear model; *R*^2^ = 0.05; *F*_year1,85_ = 3.98; *P*_year_ = 0.049).
