# Supplementary material for: High genetic diversity is not essential for successful introduction
Source: Ecol Evol. 2013 Oct 16;3(13):4501–17. doi: 10.1002/ece3.824 (PMC3856749; doi:10.1002/ece3.824)
Supplement: Supplementary file 1 [file ece30003-4501-SD1.pdf]

SUPPORTING INFORMATION

Appendix S1

Theory of adaptive genetic change in novel environments

Quantitative genetic theory predicts that the extent of adaptive genetic change (ΣGA_t) due to pre-existing genetic variation in the initial population in a new environment will be an increasing function of selection, genetic diversity, genetically effective population size (N_e) and number of generations (t), as follows (Robertson, 1960; Weber, 2004; Frankham *et al.*, 2010):

$$\sum_{i=1} \mathrm{GA}_t \sim Sh^2 \sum_{i=1} (1 - 1[2N_e]^{t-1} \qquad (1)$$

Where S is the selection differential (superiority of parents in fitness compared to the population mean, and the opportunity for selection increases with increasing fecundity), h^2 is the heritability (dependent upon the genetic variation for fitness in the new environment). Empirical results from animals and plants confirm that this is a good approximation (Weber, 2004). The predicted asymptotic genetic adaptation per generation due to new mutations since foundation of a population in the new environment is as follows (Hill, 1982b; Hill, 1982a):

$$\mathrm{GA}_{\mathrm{mutation}} \sim 2N_e S\, V_{\mathrm{m}}/V_{\mathrm{p}} \qquad (2)$$

where V_{m} is the genetic variation due to new mutations and V_{p} is the phenotypic variation. There is empirical evidence for selection response due to new mutation and it is approximately predicted by the above equation (Frankham, 1980; Frankham, 1983). In practice, the contributions of mutations to genetic adaptation are likely to become increasingly important after about twenty generations. The total genetic adaptation is predicted to be the sum of these two terms and thus in an increasing function of effective population size, selection differential, genetic diversity for fitness and the number of generations.

Appendix S2

Chromosome investigations of *Arctotheca populifolia*

Methods

Arctotheca populifolia seeds from five individuals from Australia and two individuals from South Africa were germinated in petri dishes on wet filter paper. After approximately one week of growth, seedlings had their leaves removed and the remaining roots were then pre-treated with 0.002M 8-hydroxyquinoline for 4h at room temperature. The roots were then fixed in 3:1 ethanol:acetic acid for 24h at room temperature, after which they were stored in 70% ethanol in the fridge until further use.

To prepare the slides, the roots were hydrolysed in 1M HCl for 9 min at 60°C and then stained with Schiff's reagent (Feulgen method) for 2h in the dark. In between each step, the roots were rinsed in distilled water. Squash preparations were made using root tips in a drop of 45% acetic acid. Slides were analysed with a Zeiss Axiopskop 2 MAT microscope equipped with a digital camera, and photographs were taken with Axiovision software (Zeiss).

Results and Conclusions

After viewing the chromosomes of approximately 15 specimens and 70 cells, we report that the chromosome number for the Australian and South African *Arctotheca populifolia* populations is 2n = 18. This corresponds to an earlier report (Norlindh, 1967), and is also the same number reported for one of its closest relatives, *Arctotheca calendula* (Oberprieler & Vogt, 1993). We therefore conclude that *Arctotheca populifolia* is diploid.

Table S1

Characterisation of microsatellite loci in *Arctotheca populifolia* (*N*=348) and *Petrorhagia nanteuilii* (*N*=377) including locus name, GenBank accession number, primer sequences, repeat motif, number of alleles and allele size range.

Species	Locus GenBank	Primer Sequence (5' to 3')*	Repeat Motif	Number of Alleles	Size Range*
A. populifolia	*Apo04* KC477660	F: AAAAGCTTTCGACACAGATCA R: TCTTCGTGTTTGGTAACCTGG	AC	6	92-107
A. populifolia	*Apo06* KC477661	F: GTCTTACTTCGGTTCGGCCT R: GAACGCCATCATCGTTAGGT	CT	8	214-300
A. populifolia	*Apo07* KC477662	F: ACTTTTGTTGTATTTCAAGTCCG R: ACCGAACGGTCTCAAATGTC	GA	3	88-92
A. populifolia	*Apo14* KC477663	F: GGTTTTGAACGGTCTTTGGA R: TCCTCATACTTGAGGGATTAGATATG	AG	7	217-235
A. populifolia	*Apo19* KC477664	F: ATCCGACAAGCAATTTCCAC R: TGGTCATAAGCTTCTAAATAACGAAA	TA	4	108-116
A. populifolia	*Apo33* KC477665	F: AACTGGAGTGTTGGGTCCAG R: CCAACCTGTTAAACAACCCAA	TTAT	2	182-190
A. populifolia	*Apo35* KC477666	F: CATTAGATGTTGCTGCCCCT R: CCATACTAACATGCAACCTTTG	TGTA(7)TA(3)TGTA(2)	6	148-172
P. nanteuilii	*Pna06*^ KC477667	F: TTTAGTTGAATGCGCCAACA R: TGCTTCTTGTTTTGCTTGATG	TCA	14	134-171
P. nanteuilii	*Pna07* KC477668	F: CATTTAACCCCGTGGTATCG R: AACGGTCAACCTAACCAACG	TAA	7	229-247
P. nanteuilii	*Pna09* KC477669	F: TTGTCGTTGAAATGCAGTGG R: TCATCTTGGCCGCTTAGTTC	TA	3	164-168
P. nanteuilii	*Pna16*^ KC477670	F: GTCATCCATCTTTGCCACCT R: CCCATAATATTTTCTAGGGCATCA	AAT	7	118-146
P. nanteuilii	*Pna18* KC477671	F: CATTTAGGCGTGACGGATTT R: CCAACAACACACACAACACAAG	TA	5	175-185
P. nanteuilii	*Pna22* KC477672	F: TAAATTGGACCACCAGTCCG R: AATGAGCAGTTAAGGTGGAAGC	TCT	5	79-91

**Species**	**Locus GenBank**	**Primer Sequence (5' to 3')***	**Repeat Motif**	**Number of Alleles**	**Size Range***
P. nanteuilii	*Pna24* KC477673	F: GCTTCTCAGTTATAAGGACTTGCC R: TGTTATGGAACTTGAAATAAATTTGG	TA	4	82-92
P. nanteuilii	*Pna25* KC477674	F: TGGTGGTGATCATGCAATTT R: AGGAGTCAATCACCGCTTTG	GA	2	115-119
P. nanteuilii	*Pna30* KC477675	F: ACCATTTGTAACCAACCGGA R: CATCGACACCTCTCATTCCA	AT	3	86-90
P. nanteuilii	*Pna37* KC477676	F: CGTTGCATCACATACCCAAG R: AGCTGCTGGTCGTTTTCATT	CA	5	179-189
P. nanteuilii	*Pna42* KC477677	F: CACCTAACTCGTCCTAACATCG R: ATGAATCTTAGACCATATGGAGGT	A(5)TA(8)	11	198-228
P. nanteuilii	*Pna43* KC477678	F: GTTGCCCAGCCTTTGCAT R: ACTCGGCGAAGATTGCTAAA	T(5)C(1)ATTT(7)T(4)ATTA(1)T(9)	8	98-116

* Universal primer sequence removed. ^These loci were not in Hardy-Weinberg equilibrium and were removed from downstream analyses.

Table S2

Expected heterozygosity (H_E) estimates from microsatellite data for species within the Asteraceae and Caryophyllaceae families. Effective number equivalents (^2D) have been calculated. For Asteraceae, estimates were included for one species per genus (*N*=28). For Caryophyllaceae, all estimates identified in the literature were included (*N*=8). The number of loci (L), samples (S) and total number of individuals (I) are given for each study.

Species	L	S	I	H_E	^2D	Reference
**Asteraceae**						
Achillea millefolium	16	4	25	0.39	1.64	Rahimmalek *et al.* (2011)
Ainsliaea faurieana	14	1	37	0.47	1.89	Mitsui *et al.* (2009)
Ambrosia artemisiifolia	5	10	300	0.75	4.00	Genton *et al.* (2005)
Argyroxiphium kauense	7	3	60	0.31	1.45	Friar *et al.* (2001)
Artemisia annua	8	2	54	0.41	1.69	Huang *et al.* (2009)
Aster amellus	8	3	90	0.74	3.85	Mayor & Naciri (2007)
Centaurea corymbosa	6	6	221	0.50	2.00	Freville *et al.* (2001)
Chaetanthera pusilla	7	2	50	0.67	3.03	Till-Bottraud *et al.* (2004)
Chromolaena odorata	14	1	30	0.40	1.67	Yu & Li (2011)
Cirsium acaule	6	1	25	0.59	2.44	Jump *et al.* (2002)
Crepis sancta	9	4	102	0.73	3.70	Dubois *et al.* (2007)
Cynara cardunculus	23	7	96	0.80	5.00	Khaldi *et al.* (2012)
Dubautia menziesii	7	1	30	0.22	1.28	Friar *et al.* (2006)
Echinops setifer	9	2	45	0.47	1.89	Kaneko *et al.* (2007)
Erigeron breviscapus	11	5	24	0.57	2.33	Li *et al.* (2011)
Farfugium japonicum	8	1	69	0.68	3.13	Nomura *et al.* (2009)
Hypochaeris radicata	4	17	425	0.88	8.33	Mix *et al.* (2006)
Ixeridium dentatum	7	1	32	0.59	2.44	Nakagawa & Ito (2009)
Kleinia neriifolia	17	4	96	0.70	3.33	Chen *et al.* (2012)
Launaea arborescens	10	2	42	0.44	1.79	Li *et al.* (2012)
Leontopodium japonicum	10	1	30	0.56	2.27	Lee *et al.* (2011)
Ligularia hodgsonii	14	1	30	0.54	2.17	Mao *et al.* (2009)
Lychnophora pinaster	13	2	37	0.60	2.50	Haber *et al.* (2009)
Pityopsis ruthii	12	2	20	0.57	2.33	Wadl *et al.* (2011)
Saussurea gnaphalodes	17	3	48	0.69	3.23	Zeng *et al.* (2012)
Senecio chrysanthemifolius	14	2	29	0.60	2.50	Liu *et al.* (2004)
Solidago sempervirens	9	1	96	0.68	3.13	Wieczorek & Geber (2002)
Taraxacum officinale	9	1	24	0.77	4.35	Vasut *et al.* (2004)
Mean H_E				0.58		
Arctotheca populifolia						
A_1 introduction (native range)	7	7	119	0.34	1.53	This study
A_2 introduction (native range)	7	3	69	0.07	1.09	This study

Caryophyllaceae						
Arenaria grandiflora	13	1	40	0.68	3.03	Zavodna *et al.* (2009)
Arenaria nevadensis	8	1	15	0.39	1.64	Lopez-Flores *et al.* (2008)
Lychnis flos-cuculi	7	1	18	0.83	5.88	Galeuchet *et al.* (2002)
Schiedea adamantis	10	1	49	0.35	1.72	Culley *et al.* (2008)
Silene latifolia	6	6	177	0.93	14.29	Jolivet & Bernasconi (2007)
Silene tatarica	5	8	222	0.45	1.82	Tero & Schlotterer (2005)
Silene vulgaris	7	1	45	0.82	5.56	Juillet *et al.* (2003)
Spergularia media	10	1	20	0.68	4.00	Prinz *et al.* (2009)
Mean H_E				0.64		
Petrorhagia nanteuilii						
B_2 introduction (native range)	10	2	61	0.04	1.04	This study

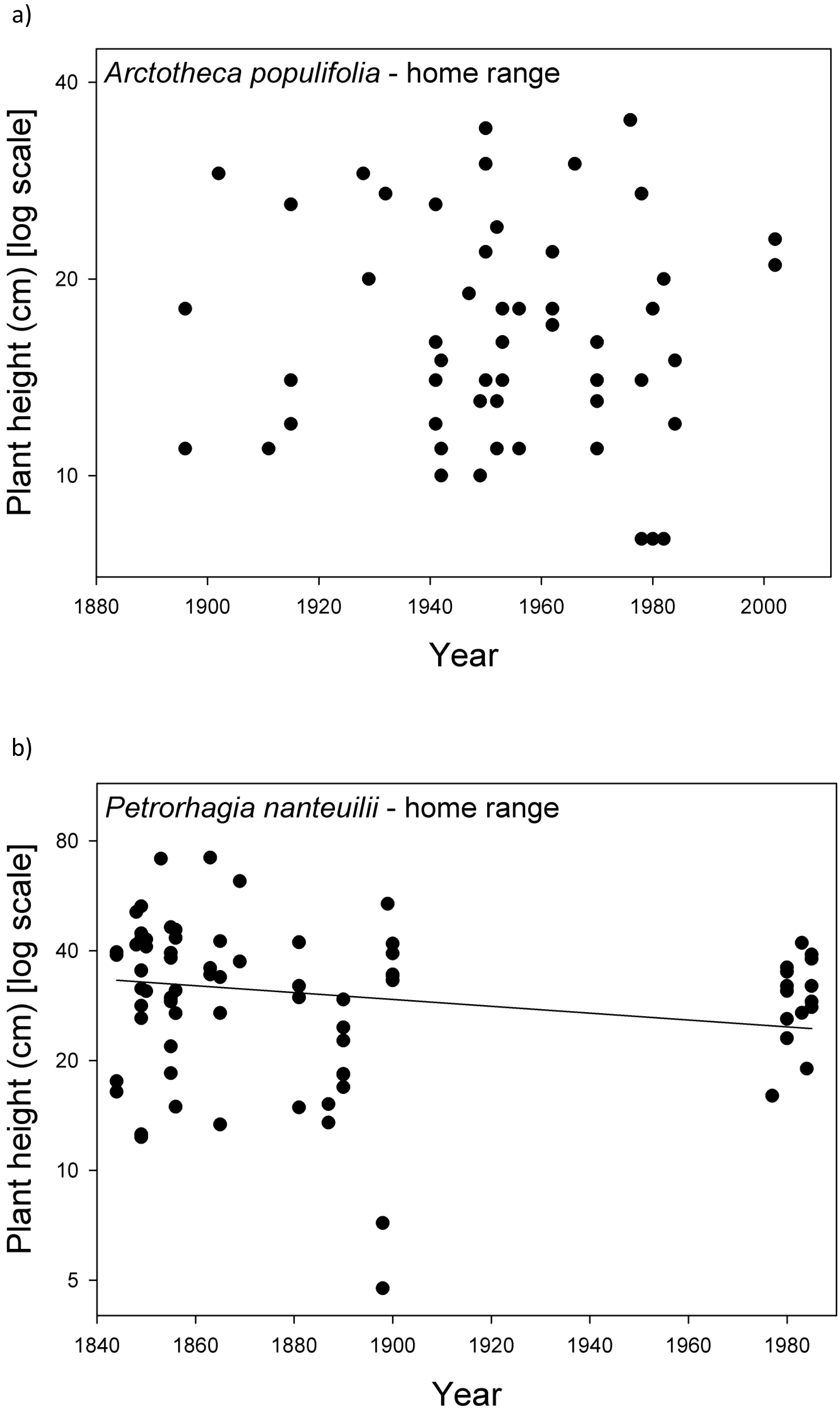

Figure S1 a and b. Plant height (log_{10} transformed) of *Arctotheca populifolia* (a) and *Petrorhagia nanteuilii* (b) measured from herbarium specimens sampled in the native range between 1891-2003 and 1848-1985, respectively. For *A. populifolia*, values do not change significantly over this time period (weighted general linear model; R^2=0.003; $F_{year1,52}$=0.15; P=0.70). For *P. nanteuilii*, plant height decreased through time (weighted general linear model; R^2=0.05; $F_{year1,85}$=3.98; P_{year}=0.049).

REFERENCES

Chen, J.-L., C. Li, et al. (2012). Microsatellite markers for *Kleinia neriifolia*, an endemic Asteraceae species on the Canary Islands. *American Journal of Botany*.

Culley, T. M., S. G. Weller, et al. (2008). Characterization of microsatellite loci in the Hawaiian endemic shrub *Schiedea adamantis* (Caryophyllaceae) and amplification in related species and genera. *Molecular Ecology Resources* **8**, 1081-1084.

Dubois, M.-P., A. Dornier, et al. (2007). Nine polymorphic microsatellite markers in *Crepis sancta* (Asteraceae). *Molecular Ecology Notes* **7**, 681-683.

Frankham, R. (1980). Origin of genetic variation in selection lines. *Selection Experiments in Laboratory and Domestic Animals*, 56-68.

Frankham, R. (1983). Origin of genetic variation in selection lines. *Proceeding of the thirty-second annual breeders' roundtable*. (ed. by R. Towner), pp. 1-18.

Frankham, R., J. D. Ballou, et al. (2010). *Introduction to Conservation Genetics,* Cambridge, Cambridge University Press.

Freville, H., F. Justy, et al. (2001). Comparative allozyme and microsatellite population structure in a narrow endemic plant species, *Centaurea corymbosa* Pourret (Asteraceae). *Molecular Ecology* **10**, 879-889.

Friar, E. A., D. L. Boose, et al. (2001). Population structure in the endangered Mauna Loa silversword, *Argyroxiphium kauense* (Asteraceae), and its bearing on reintroduction. *Molecular Ecology* **10**, 1657-1663.

Friar, E. A., L. M. Prince, et al. (2006). Ecological speciation in the east maui-endemic *Dubautia* (Asteraceae) species. *Evolution* **60**, 1777-1792.

Galeuchet, D. J., R. Husi, et al. (2002). Characterization of microsatellite loci in *Lychnis flos-cuculi* (Caryophyllaceae). *Molecular Ecology Notes* **2**, 491-492.

Genton, B. J., O. Jonot, et al. (2005). Isolation of five polymorphic microsatellite loci in the invasive weed *Ambrosia artemisiifolia* (Asteraceae) using an enrichment protocol. *Molecular Ecology Notes* **5**, 381-383.

Haber, L. H., M. M. Cavallari, et al. (2009). Development and characterization of microsatellite markers for *Lychnophora pinaster*: a study for the conservation of a native medicinal plant. *Molecular Ecology Resources* **9**, 811-814.

Hill, W. G. (1982a). Predictions of response to artificial selection from new mutations. *Genetics Research* **40**, 255-278.

Hill, W. G. (1982b). Rates of change in quantitative traits from fixation of new mutations. *Proceedings of the National Academy of Sciences* **79**, 142-145.

Huang, H.-R., G. Zhou, et al. (2009). Eight polymorphic microsatellite loci for the Chinese medicinal plant *Artemisia annua* L. (Asteraceae). *Conservation Genetics* **10**, 593-595.

Jolivet, C. & G. Bernasconi (2007). Molecular and quantitative genetic differentiation in European populations of *Silene latifolia* (Caryophyllaceae). *Annals of Botany* **100**, 119-127.

Juillet, N., H. Freymond, et al. (2003). Isolation and characterization of highly polymorphic microsatellite loci in the bladder campion, *Silene vulgaris* (Caryophyllaceae). *Molecular Ecology Notes* **3**, 358-359.

Jump, A. S., D. A. Dawson, et al. (2002). Isolation of polymorphic microsatellites in the stemless thistle (*Cirsium acaule*) and their utility in other Cirsium species. *Molecular Ecology Notes* **2**, 589-592.

Kaneko, S., Y. Isagi, et al. (2007). Development of microsatellite markers for *Echinops setifer* (Asteraceae), an endangered grassland plant species in Japan. *Conservation Genetics* **8**, 1231-1233.

Khaldi, S., G. Sonnante, et al. (2012). Analysis of molecular genetic diversity of cardoon (*Cynara carduncLulus* L.) in Tunisia. *Comptes Rendus Biologies* **335**, 389-397.

Lee, D.-H., J.-H. Lee, et al. (2011). Isolation and characterization of 10 microsatellite loci from Korean *Leontopodium japonicum* (Asteraceae). *American Journal of Botany* **98**, e183-e184.

Li, C., J.-L. Chen, et al. (2012). Development of microsatellite markers for the endangered medicinal plant *Launaea arborescens* (Asteraceae). *American Journal of Botany*.

Li, X., K. Song, et al. (2011). Isolation and characterization of 11 new microsatellite loci in *Erigeron breviscapus* (Asteraceae), an Important Chinese traditional herb. *International Journal of Molecular Sciences* **12**, 7265-7270.

Liu, G., M. J. Hegarty, et al. (2004). Isolation and characterization of microsatellite loci in *Senecio*. *Molecular Ecology Notes* **4**, 611-614.

López-Flores, I., V. Suárez-Santiago, et al. (2008). Isolation and characterization of eight polymorphic microsatellite loci for the critically endangered *Arenaria nevadensis* (Caryophyllaceae). *Conservation Genetics* **9**, 1695-1697.

Mao, C., Y. Pan, et al. (2009). Isolation and characterization of microsatellite markers for *Ligularia hodgsonii* Hook. (Asteraceae). *Conservation Genetics* **10**, 1853-1855.
Mayor, R. & Y. Naciri (2007). Identification and characterization of eight microsatellite loci in *Aster amellus* L. (Asteraceae). *Molecular Ecology Notes* **7**, 233-235.
Mitsui, Y., Y. Isagi, et al. (2009). Isolation and characterization of microsatellite loci in *Ainsliaea faurieana* (Asteraceae), an endemic plant species on Yakushima Island, Japan, and cross-species amplification in closely related taxa. *Molecular Ecology Resources* **9**, 877-879.
Mix, C., P. F. P. Arens, et al. (2006). Regional gene flow and population structure of the wind-dispersed plant species *Hypochaeris radicata* (Asteraceae) in an agricultural landscape. *Molecular Ecology* **15**, 1749-1758.
Nakagawa, S. & M. Ito (2009). Development and characterization of microsatellite loci in *Ixeridium dentatum* (Asteraceae, Lactuceae). *Journal of Plant Research* **122**, 581-584.
Nomura, N., K. Fujiwara, et al. (2009). Development and characterisation of microsatellite loci in *Farfugium japonicum* (Asteraceae). *Conservation Genetics* **10**, 1093-1095.
Norlindh, T. (1967). *Arctotheca populifolia* (Berg.) T. Norl. comb. nova. A South African dune plant. . *Aquilo Ser. Botanica* **6**, 84-93.
Oberprieler, C. & R. Vogt (1993). Chromosome numbers of North African Phanerogams II. *Willdenowia* **23**, 211-238.
Prinz, K., S. Schie, et al. (2009). Microsatellite markers for *Spergularia media* (L.) C. Presl. (Caryophyllaceae) and their cross-species transferability. *Molecular Ecology Resources* **9**, 1424-1426.
Rahimmalek, M., B. Sayed Tabatabaei, et al. (2011). Development and characterization of microsatellite markers for genomic analysis of yarrow (*Achillea millefolium* L.). *Genes & Genomics* **33**, 475-482.
Robertson, A. (1960). A theory of limits in artificial selection. *Proceedings of the Royal Society of London. Series B. Biological Sciences* **153**, 234-249.
Tero, N. & C. SchlÖTterer (2005). Isolation and characterization of microsatellite loci from *Silene tatarica*. *Molecular Ecology Notes* **5**, 517-518.
Till-Bottraud, I., T. Giraud, et al. (2004). Isolation of seven polymorphic microsatellite loci, using an enrichment protocol, in the high Andean Asteraceous *Chaetanthera pusilla*. *Molecular Ecology Notes* **4**, 462-464.
VaŠUt, R. J., P. J. Van Dijk, et al. (2004). Development and characterization of nine new microsatellite markers in *Taraxacum* (Asteraceae). *Molecular Ecology Notes* **4**, 645-648.
Wadl, P. A., A. J. Dattilo, et al. (2011). Development of microsatellite loci for the endangered species *Pityopsis ruthii* (Asteraceae). *American Journal of Botany* **98**, e342-e345.
Weber, K. (2004). Population size and long-term selection. *Plant Breeding Reviews* **24**, 249-268.
Wieczorek, A. M. & M. A. Geber (2002). Microsatellite loci for studies of population differentiation and range expansion in *Solidago sempervirens* L. (Asteraceae). *Molecular Ecology Notes* **2**, 554-556.
Yu, X.-Q. & Q.-M. Li (2011). Isolation and characterization of microsatellite markers for a worldwide invasive weed, *Chromolaena odorata* (Asteraceae). *American Journal of Botany* **98**, e259-e261.
Zavodna, M., L. Bottin, et al. (2009). Development and characterization of microsatellite markers for *Arenaria grandiflora* L. (Caryophyllaceae). *Molecular Ecology Resources* **9**, 628-630.
Zeng, L., L. Chong, et al. (2012). Microsatellite markers for *Saussurea gnaphalodes* (Asteraceae), a native Himalayan mountain species. *American Journal of Botany* **99**, e326-e329.
